# White matter abnormalities in adolescents with generalized anxiety disorder: a diffusion tensor imaging study

**DOI:** 10.1186/1471-244X-14-41

**Published:** 2014-02-15

**Authors:** Mei Liao, Fan Yang, Yan Zhang, Zhong He, Linyan Su, Lingjiang Li

**Affiliations:** 1 Department of Psychiatry, the Second Xiangya Hospital of Central South University, Changsha, China; 2 Department of Radiology, the Second Xiangya Hospital of Central South University, Changsha, China

**Keywords:** Generalized anxiety disorder, Adolescent, Diffusion tensor imaging, Fractional anisotropy

## Abstract

**Background:**

Previous neuroimaging studies have suggested an abnormal neural circuitry of emotion regulation including the amygdala and prefrontal cortex in both adult and adolescent generalized anxiety disorder (GAD) patients. Aberrant integrity of white matter in this neural circuitry has been verified in adult GAD patients. White matter abnormalities in adolescent GAD patients have not been detected.

**Methods:**

Twenty-five adolescents with GAD and 24 healthy controls underwent a diffusion tensor imaging scan. Fractional anisotropy (FA) was compared between groups with a voxel-wise Tract-Based Spatial Statistics (TBSS) analysis method.

**Results:**

Compared with healthy controls, adolescents with GAD showed significantly reduced FA in bilateral uncinate fasciculus, inferior fronto-occipital fasciculus, inferior longitudinal fasciculus, and corona radiata.

**Conclusions:**

The findings in the present study suggest a neural basis of emotion dysregulation in adolescent GAD patients.

## Background

Generalized anxiety disorder (GAD) is a common anxiety disorder with an estimated lifetime prevalence of 5.7% in the general population [1]. This condition is characterized by chronic and excessive worry about everyday things and often begins in adolescence or early adulthood [2]. GAD usually increases the risk of other anxiety disorders and depressive disorder [2] and causes significant distress or impairment in life [3]. However, compared with other anxiety disorders, GAD is less studied [4], despite its high prevalence and clinical importance.

Neuroimaging approaches have provided promising avenues for studying the underlying neural circuitry of GAD. Previous neuroimaging studies have suggested an abnormal neural circuitry of emotion regulation including the amygdala and prefrontal cortex in both adult and adolescent GAD patients [5-7]. The amygdala is a key component in the neural circuit, processing emotional valence and generating rapid affective responses [8,9]. Functional neuroimaging studies have shown altered activation in the amygdala. Investigations on pediatric and adolescent GAD patients as well as adult GAD patients have exhibited increased amygdala activation to threat stimuli [7,10-12]. Some studies on adult GAD patients have demonstrated decreased activity in the amygdala during a gambling game [13] or in response to threat stimuli [14] in GAD patients compared to healthy controls. Structural neuroimaging studies showed larger gray matter volumes in the amygdala in adult [15,16] and adolescent [17] GAD patients, whereas one study on adolescent GAD samples yielded decreased amygdala volumes [18].

The prefrontal cortex, including the anterior cingulate cortex, is involved in emotional regulation by down-regulating the activity of the amygdala and related limbic structures [5,19]. Functional neuroimaging studies of adolescents with GAD have shown increased activation in the lateral prefrontal cortex [10,20,21], medial prefrontal cortex [21] and anterior cingulate cortex [10] while viewing noxious stimuli. One study [14] found that adult GAD patients exhibited increased activation to angry expressions in the lateral frontal cortex, whereas another study [22] suggested that adult GAD patients showed decreased activation to emotional conflict in the dorsomedial prefrontal cortex compared with healthy controls. The limited structural neuroimaging studies have shown a sub-threshold decrease of gray matter volume in bilateral ventrolateral cortex in pediatric anxiety patients [18], and a larger volume of the dorsomedial prefrontal cortex in adult GAD patients [15]. In addition, a functional connectivity study [22] has revealed the aberrant functional connection between amygdala and the prefrontal cortex, suggesting neural correlates for emotion regulation deficits in adults with GAD. Taken together, all the available neuroimaging evidence suggests an abnormality of the neural circuitry, including the amygdala and prefrontal cortex, in both adult and adolescent GAD patients.

Most studies investigate abnormities of gray matter, while few focus on the integrity of white matter. Diffusion tensor imaging (DTI) techniques can be used to examine the structural integrity of white matter and to map white matter tracts by measuring the magnitude and direction of water diffusion [23]. Two studies [24,25] found that adult GAD patients showed lower fractional anisotropy (FA) in the uncinate fasciculus that is located at the frontal lobe and connects the amygdala and prefrontal cortex. Recently, a study by our group [26] suggested that adult GAD patients had higher FA in the right amygdala white matter and lower FA in the caudal anterior cingulate cortex white matter compared with healthy controls. These three studies have also suggested an abnormal neural circuitry of emotion regulation, including the amygdala and prefrontal cortex, in adult GAD patients. However, subjects in these three studies are adult. To the best of our knowledge, no study has reported on white matter integrity in adolescent GAD patients. Therefore, the primary purpose of this study was to investigate the possible abnormal integrity of white matter in adolescents with GAD. Based on the evidence described earlier, we hypothesized that adolescent GAD patients would show aberrant white matter integrity in the neural circuitry associated with emotion regulation.

## Methods

### Subjects

This study was approved by the Ethics Committee of the Second Xiangya Hospital of Central South University, China. Written informed consent was obtained from each adolescent and one of his or her legal guardians after the study had been fully explained. Twenty-five adolescents with GAD (13 girls, 12 boys) and 24 healthy controls (11 girls, 13 boys) participated in the present study. All subjects were recruited from local high schools in Hunan Province via advertisements and school notice. First, 1885 subjects finished the 41-item self-report questionnaire, the Screen for Child Anxiety Related Emotional Disorders (SCARED) [27,28]. The SCARED is a reliable and valid screening tool for childhood anxiety disorder, with an optimal total cutoff point score of 25 to separate children with anxiety disorders from those without [27,28]. Among 1885 subjects, 508 subjects’ SCARED scores were greater than 25, and the scores of the rest were lower than 25. Then, 673 subjects (508 SCARED scores ≥ 25; 165 SCARED scores < 25) were investigated by the same trained clinician and diagnosed using DSM-IV criteria and the Schedule for Affective Disorders and Schizophrenia for School Age Children-Present and Lifetime (K-SADS-PL) version [29]. Inclusion criteria for patients in this study were current first-episode, generalized anxiety disorder without co-morbidity disorders. Healthy controls met criteria for no mental disorders. Exclusion criteria for all subjects included any neurological abnormalities, history of seizures, head trauma or unconsciousness, any physical disease and use of psychoactive substances. All subjects enrolled in this study were non-medicated, right-handed, and volunteered to participate in this study. In addition, all the patients were assessed with the Penn State Worry Questionnaire (PSWQ) [30] , which was introduced to assess anxiety levels of adolescent GAD patients.

### Diffusion tensor imaging data acquisition

Diffusion tensor imaging was performed using a 3.0 Tesla Philips scanner, equipped with a SENSE-8 channel head coil, at the Second Xiangya Hospital of Central South University, China. For each participant, images were acquired using a single shot spin echo-echo plane sequence in alignment with the anterior-posterior commissural plane with the following parameters: repetition time = 6590 milliseconds, echo time = 70 milliseconds, acquisition matrix = 128 × 128, field of view = 240 mm × 240 mm, flip angle = 90°, slice thickness = 2.5 mm without gap. The diffusion sensitizing gradients were applied along 33 non-collinear directions (b = 1000 s/mm^2^), together with an acquisition without diffusion weighting (b = 0 s/mm^2^).

### Image data processing

The processing of DTI data was conducted with Tract-Based Spatial Statistics (TBSS) version 1.2 implemented in the FMRIB Software Library (FSL version 5.0, Oxford, Untied-Kingdom; http://www.fmrib.ox.ac.uk/fsl), according to the standard procedure described in detail in the previous study [31]. First, DTI data were preprocessed to create FA maps. All images were corrected for the effects of head movement and eddy currents using FMRIB’s Diffusion Toolbox (FDT) [32] in FSL. A brain mask was created from the b0 image by running Brain Extraction Tool (BET) [33] and FA maps were calculated by fitting a tensor model to the raw diffusion data [32]. Then, the resulting FA maps were further analyzed using TBSS [31]. In general, FA maps of all subjects were aligned into a common (Montreal Neurological Institute 152 standard) space using the nonlinear registration tool FNIRT. The transformed FA images were averaged to create a mean FA image, and the tracts were thinned to create a mean FA skeleton that represents the centers of all tracts common to the group. The FA threshold was set at 0.2 to confine the analysis to white matter. Each subject’s FA image was projected onto this skeleton and the resulting data fed into voxel-wise between-subject statistics.

### Statistical analysis

Statistical analysis for the demographic and clinical measures was conducted with two-sample *t* tests or chi-square tests, as needed, in SPSS16. The threshold for statistical significance was p < 0.05. A voxel-wise statistical analysis, with age as a covariant, was performed to explore regions of significant differences of FA images between GAD patients and healthy controls using the randomize tool in FSL. The contrast was computed using 5000 permutations [34]. The results were corrected for multiple comparisons using the threshold-free cluster enhancement (TFCE) approach, which allowed us to avoid making an arbitrary choice of the cluster-forming threshold, while preserving the sensitivity benefits of cluster-wise correction. The threshold for significance was set at p < 0.05. The Johns Hopkins University International Consortium for Brain Mapping (JHU ICBM)-DTI-81 white-matter atlas [35] and JHU white-matter tractography atlas [36] labels were used to label significant voxels and assign a specific tract name. For regions of significant differences, FA values were extracted from each participant’s FA image. Correlation analyses was conducted to investigate the possible association between the severity of clinical measures (PSWQ scores) and white matter integrity (FA values). The level of two-tailed statistical significance was set at P < 0.05.

## Results

There were no significant differences in age (GAD group mean age 16.96 ± 0.68 years; control group mean age 16.58 ± 0.83 years; p = 0.087) or gender (GAD group 13 girls/12 boys; control group 11 girls/13 boys; p = 0.778) between the two groups (Table 1).

**Table 1:** Demographic and clinical measures of adolescent GAD patients and healthy controls

	**GAD (25)**	**HCs (24)**	**Statistical value**	***P***
Age, mean ± SD, year	16.96 ± 0.68	16.58 ± 0.83	1.746	0.087
Gender, No, girls/boys	13/12	11/13	0.666	0.778
PSWQ score, mean ± SD	54.64 ± 8.85	--	--	--

*GAD*, Generalized anxiety disorder; *HCs*, Healthy controls; *PSWQ*, The Penn State Worry Questionnaire.

**Figure 1:**
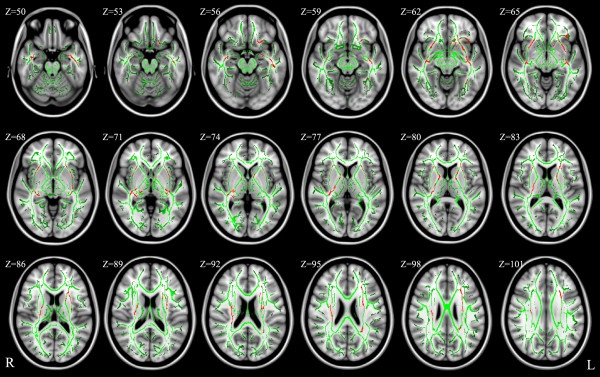
**Results from TBSS analysis showing the regions of significant FA reduction in adolescent GAD patients compared with healthy controls, (p < 0.05, TFCE-corrected).** Voxels are overlaid on the white matter skeleton (green). The regions of significant FA reduction are shown in red. The background images are MNI152 template. MNI, Montreal Neurological Institute; L, left; R, right.

The results of voxel-wise analyses in TBSS are shown in Figure 1 and Table 2. Adolescent GAD patients had significantly reduced FA in bilateral uncinate fasciculus, inferior fronto-occipital fasciculus, inferior longitudinal fasciculus, and corona radiata (p < 0.05, TFCE-corrected). In addition, significantly reduced FA also were found in the left anterior limb of internal capsule and superior longitudinal fasciculus, as well as the right posterior limb of internal capsule (p < 0.05, TFCE-corrected). There were no white matter regions where the controls had significantly lower FA compared with the GAD patients.

**Table 2:** Regions of reduced white matter FA in adolescent GAD patients compared with healthy controls (p < 0.05, TFCE-corrected)

**Cluster index**	**Anatomic location**	**L/R**	**Cluster size**	**Peak coordinates**		
				**x**	**y**	**z**
1	Uncinate fasciculus, Inferior fronto-occipital fasciculus, Inferior longitudinal fasciculus, Superior corona radiate, Anterior limb of internal capsule, Superior longitudinal fasciculus	L	2778	−40	−15	−13
2	Uncinate fasciculus, Inferior fronto-occipital fasciculus, Inferior longitudinal fasciculus, Posterior corona radiate, Posterior limb of internal capsule	R	1766	28	−17	23
3	Uncinate fasciculus, Inferior fronto-occipital fasciculus	R	346	30	11	−8
4	Inferior fronto-occipital fasciculus, Posterior corona radiata	L	187	−33	−50	12
5	Uncinate fasciculus, Inferior fronto-occipital fasciculus	L	187	−38	17	−15
6	Inferior longitudinal fasciculus, Anterior corona radiate	L	130	−27	−50	20
7	Inferior longitudinal fasciculus, Inferior fronto-occipital fasciculus	L	111	−31	−43	−16

*FA*, Fractional anisotropy; *GAD*, Generalized anxiety disorder; *TFCE*, The threshold-free cluster enhancement; *L/R*, Left/right.

Correlation analyses revealed no significant relationship between PSWQ scores and FA values of regions of significant differences (p > 0.05).

## Discussion

In the present study, we used DTI data to compare the integrity of white matter between adolescent GAD patients and healthy controls through whole brain voxel-wise TBSS analysis. To the best of our knowledge, this is the first study to explore abnormalities of white matter integrity in adolescent GAD patients. The result of reduced FA in the uncinate fasciculus is consistent with previous studies in adult GAD patients [24,25]. A monozygotic twin study [24] with 17 pairs of adult females discordant for lifetime GAD revealed that the affected twins showed reduced FA values in the uncinate fasciculus. Another study [25] compared 49 adult patients with GAD and 39 healthy controls and found that patients with GAD had decreased mean FA values in the bilateral uncinate fasciculus. The uncinate fasciculus plays an important role in connecting the prefrontal cortex and anterior cingulate cortex to the amygdala and other limbic areas. The abnormal integrity of the uncinate fasciculus, therefore, would be associated with abnormal activation of the prefrontal cortex, anterior cingulate cortex, and amygdala.

The reduced FA values of the uncinate fasciculus in this study might suggest decreased negative association between prefrontal cortex and the limbic structures, which would induce deficits in emotion regulation and cause anxiety. The abnormal integrity of uncinate fasciculus in the present study is in consistent with previous functional neuroimaging studies [7,10-12,20-22]. Increased activation of the amygdala [7,10-12], prefrontal cortex [10,20,21], and the anterior cingulate cortex [10] in GAD patients compared with healthy controls has been reported in these previous studies when subjects were involved in emotion regulation or conflict monitoring and viewing emotion stimuli. In addition, Etkin et al. [22] conducted a functional connectivity analysis and found abnormal functional connection between the amygdala and the prefrontal cortex in GAD patients. Hence, some authors have suggested that the reason for hyperactivity of the amygdala, prefrontal cortex, and anterior cingulate cortex in GAD patients compared with healthy controls is the decreased coupling between these brain regions [22,25], which might be associated with the reduced FA values of the uncinate fasciculus.

Our results also suggested reduced FA in bilateral inferior fronto-occipital fasciculus, which spans from the frontal lobe to the occipital lobe by passing through the temporal lobe. The inferior fronto-occipital fasciculus might connect the frontal lobe and occipital lobe and modulate anxiety responses to environment stimulus [37]. Decreased FA in the inferior fronto-occipital fasciculus has been reported in other anxiety disorders, i.e., panic disorder [38], and obsessive compulsive disorder [39]. Reduced FA in the inferior fronto-occipital fasciculus might decrease the linking between the occipital lobe and the frontal lobe, and disturb sensory integration and cognitive or emotion regulation to sensory stimulus.

Compared with healthy controls, reduced FA in the inferior longitudinal fasciculus also was found in adolescent GAD patients, which is in consistent with the study conducted by Hettema et al. [24], who found decreased FA in the inferior longitudinal fasciculus in adult GAD twins. The inferior longitudinal fasciculus, connecting occipital and temporal lobe structures including the amygdala, hippocampus, and parahippocampus, is an important component of the visual limbic pathway that subserves emotional, learning and memory functions [40]. It is suggested that alterations of white matter integrity in the inferior longitudinal fasciculus might be associated with anxiety state by abnormal perception of environment visual information [40]. In this study, we also found decreased FA in bilateral corona radiata, left anterior limb of internal capsule, and right posterior limb of internal capsule, which include descending sensorimotor fibers contributing to the corticospinal tract [41]. The fiber tracts in the corona radiata and internal capsule, reciprocally project from the cortex to the thalamus and pontine nuclei [42]. The thalamus plays an important role in filtering sensory information and emotional regulation [43]. Alterations in the corona radiata and internal capsule might disturb the connection between thalamus and cortex, and induce abnormal emotional regulation. More evidence is needed to verify these findings.

Some limitations of this study should be acknowledged. The sample size of the present research was modest, which might result in type II errors and decrease true positive effects. A larger sample is required in future investigations. We only studied adolescent GAD patients aged 16 to 18, which might restrict the generalization of our findings. Investigations of different age stages in adolescence are required to confirm the results in our study. We excluded GAD patients with comorbidities in this investigation. GAD often co-occurs with other mental disorders [2,3], such as other anxiety disorders and major depression disorder. The homogenous sample allows us to draw a more specific conclusion on GAD; however, it also limits the generalization of our findings. Further research is needed to specify the possible aberrant white matter integrity in GAD patients with and without comorbidities, respectively.

## Conclusions

In conclusion, this is the first study to investigate whole-brain white matter integrity in adolescent GAD patients. We found significantly reduced FA in bilateral uncinate fasciculus, inferior fronto-occipital fasciculus, inferior longitudinal fasciculus, and corona radiata in adolescents with GAD compared with healthy controls in the current study. Our results suggested aberrant white matter integrity in the neural circuitry associated with emotion regulation in adolescent GAD patients. This study provides a possible neurobiological basis for adolescent GAD patients. In addition, further investigation is required to replicate and confirm the results in our study.

## Competing interests

The authors declare that they have no competing interests.

## Authors’ contributions

Author ML designed this study and wrote the first draft of the manuscript. Author FY recruited the sample and finished the clinical assessment. Author YZ managed the data analysis. Author ZH completed MRI scanning. Authors LL and LS also designed the study and had full access to all of the data in the study and take responsibility for the integrity of the data and the accuracy of the data analysis. All authors contributed to and have approved the final manuscript.

## Pre-publication history

The pre-publication history for this paper can be accessed here:

http://www.biomedcentral.com/1471-244X/14/41/prepub
